# Matrine Inhibits Infiltration of the Inflammatory Gr1^hi^ Monocyte Subset in Injured Mouse Liver through Inhibition of Monocyte Chemoattractant Protein-1

**DOI:** 10.1155/2013/580673

**Published:** 2013-08-22

**Authors:** Duo Shi, Jinjin Zhang, Lei Qiu, Jianzhong Li, Zhenlin Hu, Junping Zhang

**Affiliations:** Department of Biochemical Pharmacy, School of Pharmacy, Second Military Medical University, 325 Guohe Road, Shanghai 200433, China

## Abstract

Matrine (Mat) is a major alkaloid extracted from *Sophora flavescens* Ait, an herb which is used in the traditional Chinese medicine for treatment of inflammation, cancer, and other diseases. The present study examined the impact of Mat on the CCl_4_-induced hepatic infiltration of Gr1^hi^ monocytes to explore the possible mechanisms underlying its anti-inflammatory and antifibrotic effects. The results indicated that Mat protected mice from acute liver injury induced by single intraperitoneal injection of CCl_4_ and attenuated liver fibrosis induced by repeated CCl_4_ injection. Meanwhile, the infiltrations of Gr1^hi^ monocytes in both acute and chronic injured livers were all inhibited, and the enhanced hepatic expression of MCP-1 was suppressed. Cellular experiments demonstrated that Mat directly inhibited MCP-1 production in both nonparenchymal cells and hepatic stellate cells derived from CCl_4_-injured livers. Transwell chemotaxis assays showed that Mat significantly inhibited the chemotactic activity of MCP-1. These results suggest that the anti-inflammatory and antifibrotic effects of Mat could be contributed, at least in part, to its prevention of Gr1^hi^ monocyte infiltration into the injured livers and inhibition of MCP-1 production and activity. These findings extend our understanding of the mechanisms underlying the anti-inflammatory and antifibrotic effects of Mat.

## 1. Introduction

Chronic hepatic injury may lead to liver fibrosis [1]. After an acute liver injury, parenchymal cells regenerate and replace dead cells. This process is associated with an inflammatory response and a limited deposition of extracellular matrix (ECM). If hepatic injury persists, liver regeneration eventually fails and hepatocytes are substituted with abundant ECMs. Activated hepatic stellate cells (HSCs), portal fibroblasts and myofibroblasts, have been identified as major ECM-producing cells in injured livers [2]. Activation of liver-resident macrophages, the so-called Kupffer cells (KCs), has been indicated as an initial event in the process leading to liver injury and fibrosis caused by different etiologies [3]. It is well established that activated KCs play an important role in perpetuating an inflammatory phase resulting in the massive release of proinflammatory and fibrogenic mediators as well as activation of HSCs [4–7]. However, recent studies demonstrate that these actions are only partially conducted by KCs, but they largely depend on recruitment of monocytes into the livers [8, 9].

Blood monocytes are circulating precursors of tissue macrophages. Macrophages and monocytes are characterized by lack of lymphocyte markers and by expression of CD11b and CD14 in humans and of CD11b and F4/80 in mice [10–12]. Murine monocytes can be subdivided by their expression of Gr1 and of the chemokine receptors CCR2 and CX3CR1. Gr1^hi^ monocytes express high levels of C-C chemokine receptor CCR2 but lack CX3CR1, whereas Gr1^lo^ monocytes lack CCR2 but express high levels of CX3CR1. Their counterparts in humans are CD14^++^CD16^−^CCR2^+^ and CD14^+^CD16^+^CCR2^−^ monocytes, respectively. Gr1^hi^ monocytes actively enter inflamed tissue and are considered precursors for macrophages and dendritic cells in inflammatory conditions, whereas Gr1^lo^ monocytes home to noninflamed tissues and may represent steady-state precursor cells for tissue macrophages [12, 13]. Differential recruitment of these monocyte subsets appears to be crucially controlled by chemokine released from injured tissue. It has been suggested that CCR2 mediates entry of inflammatory Gr1^hi^ monocytes into inflamed tissues [14–21]. More importantly, enhanced hepatic expression of monocyte chemoattractant protein-1 (MCP-1), a specific ligand of CCR2, has been shown to contribute to the formation and maintenance of inflammatory infiltrate during chronic liver disease [22].

A more recent study further demonstrates that inflammatory Gr1^hi^ but not Gr1^lo^ monocytes are massively recruited into the carbon tetrachloride-(CCl_4_-) injured livers in a CCR2-dependent manner [18]. Furthermore, hepatic Gr1^hi^ monocyte-derived cells in CCl_4_-injured livers exert proinflammatory and pro-fibrogenic actions, such as promoting HSC activation, TH1 cell differentiation, and TGF-*β* release, during liver fibrogenesis. Impaired monocyte subset recruitment in CCR2-deficient mice reduces HSCs activation and diminishes liver fibrosis. Moreover, adoptively transferred Gr1^hi^ monocytes traffick into the injured livers and promote fibrosis progression in wild-type and CCR2-deficient mice [18]. These experiments provide evidence for a vital role of MCP-1/CCR2-dependent Gr1^hi^ monocytes infiltration in the development of liver fibrosis upon hepatic injury, thus suggesting that modulation of monocyte subset recruitment into liver may represent an approach for antifibrotic strategy.

The herbal medicine Kushen consists of the dried roots of *Sophora flavescens* Ait. It was first described in *Shen Nong Ben Cao Jing* in 200 A.D. as a treatment for inflammation, solid tumors, and many other diseases. In the traditional Chinese medicine, Kushen is commonly used as decoction or powder of dried roots for treatment of a wide variety of conditions including viral hepatitis, cancer, enteritis, viral myocarditis, arrhythmia, colpitis, and eczema [23]. Kushen alkaloids are considered to be its major active components and have been widely used in China for the treatment of hepatitis and cancers. As one of the major Kushen alkaloids, Matrine (Mat) has been demonstrated to posses significant anti-inflammatory and antifibrotic properties [24–34]. However, the underlying mechanisms have not been fully elucidated. In the present study, we investigated whether Mat could modulate the recruitment of Gr1^hi^ monocytes into CCl_4_-injured liver in mice. We herein demonstrated that Mat protected mice against CCl_4_-induced hepatic injury and prevented infiltration of inflammatory Gr1^hi^ monocytes into the injured livers, possibly through inhibiting the production and activity of MCP-1. These new findings extend our understanding of the mechanisms underlying the anti-inflammatory and antifibrotic effects of Mat.

## 2. Materials and Methods

### 2.1. Chemicals and Reagents

Mat (purity > 99%) was purchased from National Institute for the Control of Pharmaceutical and Biological Products, China. Recombinant mouse TNF-*α* and MCP-1 were purchased from PeproTech (Rocky Hill, NJ, USA). Lipopolysaccharide (LPS) and DMEM medium were purchased from Sigma (St. Louis, MO, USA).

### 2.2. Mice

C57BL/6 mice (6-week-old, females) were purchased from Shanghai SLAC Laboratory Animal Co., Ltd. (Shanghai, China). All experiments were performed in accordance with the Institutional Animal Care Instructions approved by the Ethics Committee for Animals of the Second Military Medical University.

### 2.3. CCl_4_-Induced Acute Hepatic Injury Model

C57BL/6 mice were injected i.p. with CCl_4_ (0.6 mL/kg body weight, diluted 1 : 3 in corn oil) to induce acute hepatic injury. As normal control, animals received the same volume of corn oil intraperitoneally. To test the protective effect of Mat, mice (*n* = 8/group) were administrated orally either with Mat (10 mg/kg, 30 mg/kg) in 0.2 mL of PBS or with the same volume of PBS, 3 h prior to CCl_4_ injection.

### 2.4. CCl_4_-Induced Chronic Hepatic Injury Model

C57BL/6 mice were repeatedly injected i.p. with CCl_4_ (0.6 mL/kg body weight) twice weekly for 6 weeks to induce chronic liver injury. To test the protective effect of Mat, mice (*n* = 8/group) were administrated orally either with Mat (10 mg/kg) in 0.2 mL of PBS or with the same volume of PBS, 5 times weekly for last three weeks. Mice were sacrificed 48 h after the the last CCl_4_ injection.

### 2.5. Measurement of Serum Alanine Aminotransferase (ALT)

Blood samples were collected at the indicated time points after CCl_4_ injection, and serum ALT levels were measured using a colorimetric assay kit (Nanjing Jiancheng Bioengineering Institute, Nanjing, China) according to the manufacturer's protocol.

### 2.6. Hydroxyproline Assay

Hepatic content of hydroxyproline was determined using the Hydroxyproline Testing Kit (Jiancheng, Nanjing, China) according to the manufacturer's protocol.

### 2.7. Histological Analysis

The mouse livers were removed at the indicated time points after CCl_4_ challenge. Liver samples were fixed in 10% formalin solution, embedded in paraffin, and sectioned. Hematoxylin-eosin, Sirius red, and Masson staining, were performed according to standard protocols. For immunohistochemical analysis, liver specimens were fixed in 4% paraformaldehyde, and immunohistochemical staining was performed according to standard procedures using monoclonal hamster anti-mouse CCL2/MCP-1 antibody (BioLegend, San Diego, CA, USA) as the primary antibody and horseradish-peroxidase-conjugated goat anti-armenian hamster IgG (Santa Cruz Biotechnology, Inc., CA, USA) as the secondary antibodies. Sections were counterstained with hematoxylin and evaluated by light microscopy.

### 2.8. Isolation of Nonparenchymal Cells (NPCs) and HSCs

NPCs were isolated as described previously [35]. Briefly, under ether anesthesia, the peritoneal cavity was aseptically exposed, and the inferior vena cava was cannulated. Liver was perfused *in situ* first with 50 mL of 1x Hank's balanced salt solution (pH 7.4, 37°C), followed by perfusion with 1% collagenase type IV (Sigma-Aldrich) solution until the hepatic parenchyma beneath the capsule appeared liquefied (approximately for 5 min). After hepatectomy, the liver was transferred to a Petri dish containing 10 mL DMEM medium and was gently minced. This slurry was then filtered (mesh size 70 *μ*m) to remove large aggregates. Low-speed (30 g) centrifugation of the liver suspension was performed to exclude hepatocytes, followed by high-speed (500 g) centrifugation to obtain NPCs. After 3 washes, the NPCs were used for flow cytometry analysis or *in vitro *culture for cytokines induction examination. HSCs were enriched from NPCs by centrifugation over a 3-layer Percoll (GE Healthcare) gradient (52%, 50%, and 30%) as described previously [36]. After centrifugation, HSCs were collected from the interface, washed with Hank's balanced salt solution, and resuspended in DMEM medium supplemented with fetal bovine serum (FBS) (20%), penicillin (100 U/mL), and streptomycin (100 U/mL).

### 2.9. Flow Cytometry Analysis of NPCs

Expression levels of various cell surface antigens on NPCs were analyzed by flow cytometry on a FACS Calibur flow cytometer (BD Biosciences, San Jose, CA, USA) using combinations of fluorochrome-conjugated mAbs against CD45, CD11b, F4/80, or Gr1. All mAbs used in this study were purchased from BioLegend (San Diego, CA, USA).

### 2.10. *In Vitro* Induction of MCP-1 in NPCs and HSCs

Freshly isolated NPCs were suspended in DMEM medium supplemented with FBS (10%), penicillin (100 U/mL) and streptomycin (100 U/mL), and wereplated onto 12-well plates at 1 × 10^6^ cells/well. After 24 h incubation at 37°C, 5% CO_2_, NPCs attached to plates were stimulated with 1 *μ*g/mL of LPS for 24 h in the absence or presence of Mat at concentrations of 10, 20, 50, or 100 *μ*M. Levels of MCP-1 in supernatants were quantified with commercial mouse ELISA kit (R&D Systems, Inc., MN, USA) according to the manufacturer's instructions.

Freshly isolated HSCs were suspended in DMEM medium supplemented with FBS (20%), penicillin (100 U/mL), and streptomycin (100 U/mL), plated onto 6-well plates at 5 × 10^6^ cells/well, and was allowed to attach to plates by incubation overnight. Then, the culture medium was replaced with fresh DMEM supplemented with FBS (0.5%), penicillin (100 U/mL), and streptomycin (100 U/mL), and the cells were cultured for another 24 h. TNF-*α* (30 ng/mL) was added to the medium in the absence or presence of different concentrations of Mat (10, 20, 50, or 100 *μ*M). After 24 h incubation, cellular mRNA levels of MCP-1 were measured by quantitative real-time PCR.

### 2.11. Quantitative Real-Time PCR

Total RNA was extracted from HSCs or liver tissues with the TRIzol reagent (Invitrogen), and it was reverse transcribed with a complementary DNA reverse-transcription kit (Takara, Dalian, China) according to the manufacturer's instructions. Quantitative real-time PCR was performed on a StepOnePlus real-time PCR system (Applied Biosystems, USA) using the SYBR Premix Ex Taq PCR Kit (Takara, Dalian, China). The primers used were designed and custom synthesized at Invitrogen. The relative levels of assayed mRNAs were calculated with the comparative CT method using GAPDH expression levels as endogenous control, and they were normalized to nontreated control. The primers used were

5′-CTTCTGGGCCTGCTGTTCACAGTT-3′ (MCP-1 forward), 5′-TTCTTGGGGTCAGCACAGACCTCT-3′ (MCP-1 reverse) and 5′-ATCTTCTTGTGCAGTGCCAGCCTC-3′ (GAPDH forward), 5′-TTTGCCACTGCAAATGGCAGCC-3′ (GAPDH reverse).

### 2.12. Transwell Chemotaxis Assays

Peripheral blood mononuclear cells (PBMCs) were isolated from heparinized murine blood by density separation over Ficoll-Hypaque. The blood was layered on top of the Ficoll-Hypaque at a 2 : 1 ratio in 15 mL tubes and centrifuged for 25 minutes at 2500 rpm at room temperature. PBMCs at the interface were carefully collected and washed twice in HBSS. 

Transwell chemotaxis assays were performed in 24-well transwells (5 *μ*m pore size; Costar, Cambridge, MA, USA). 1 × 10^5^ PBMCs in 0.1 mL of medium were added to the upper chamber of 2 compartments in the absence or presence of Mat at various concentrations. 50 ng/mL of MCP-1 in 0.6 mL of medium was added to the lower compartment. After incubating the plate at 37°C for 2 h, the nonmigrating cells were removed from the upper surface of the membrane. The cells on the lower surface of the membrane were fixed with ice-cold methanol and then stained with crystal violet. The number of migrated cells was counted with microscopy.

### 2.13. Statistical Analysis

 All quantitative data are expressed as the mean ± (SD). Statistical analysis was performed using one-way analysis of variance (ANOVA) test, followed by Dunnett's multiple-comparison post hoc test. *P* < 0.05 was considered to be statistically significant.

## 3. Results

### 3.1. Mat Protects Mice against CCl_4_-Induced Acute Liver Injury and Reduces Hepatic Inflammatory Infiltratation

**Figure 1:**
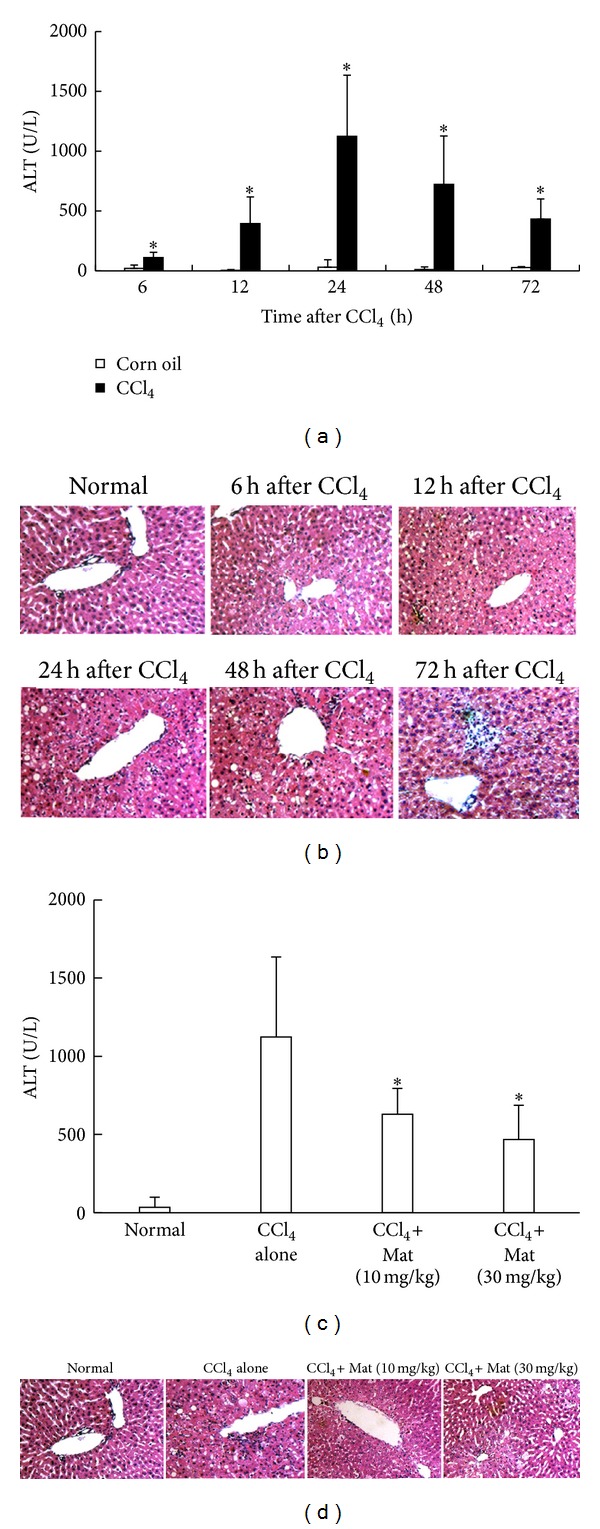
Mat protected mice against CCl_4_-induced acute liver injury and reduced hepatic inflammatory infiltration. (a) C57BL/6 mice were injected i.p. with CCl_4_ (0.6 mL/kg) diluted in corn oil, and sera were collected and analyzed for ALT levels at indicated time points. Data are expressed as the mean ± (SD) (*n* = 8 mice/group). *P < 0.05 versus corn oil control. (b) Liver tissues were collected at indicated time points after CCl_4_ injection, and liver sections were made and stained with hematoxylin-eosin (HE) (original magnification ×200). (c) Mat (10 mg/kg, 30 mg/kg) was administrated orally 3 h before CCl_4_ injection, and sera were collected and analyzed for ALT levels at 24 h after CCl_4_ injection. Data are expressed as the mean ± (SD) (*n* = 8 mice/group). ^#^ *P* < 0.05 versus normal group; *P < 0.05 versus CCl_4_-alone group. (d) Liver tissues were collected 24 h after CCl_4_ injection, and liver sections were made and stained with HE (original magnification ×200).

The present study was initiated by investigating the protective effect of Mat on CCl_4_-induced acute liver injury in mice. As shown in Figure 1, single intraperitoneal injection of CCl_4_ resulted in a time-dependent increase in serum ALT levels. Significant increases in serum ALT levels were detected at 6 h after CCl_4_ injection. Serum ALT levels peaked at approximately 24 h, and they then started to subside by 48 h in CCl_4_-challenged mice (Figure 1(a)). Histological examination of liver sections from CCl_4_-challenged mice showed that CCl_4_ resulted in periportal necrosis with maximal damage at 24 h, and toxic damage was accompanied by a massive infiltration of leukocytes into the liver (Figure 1(b)). Mice treated with 10 mg/kg or 30 mg/kg of Mat 3 h prior to CCl_4_ challenge showed significant decrease in ALT levels at 24 h after CCl_4_-injection (Figure 1(c)). Treatment with Mat also markedly attenuated pathologic changes in CCl_4_-challenged mice. It is important to note that Mat treatment resulted in a dramatic decrease in hepatic inflammatory infiltration in CCl_4_-challenged mice (Figure 1(d)).

### 3.2. Mat Inhibits CCl_4_-Induced Hepatic Infiltration of Gr1^hi^ Monocytes Subset

**Figure 2:**
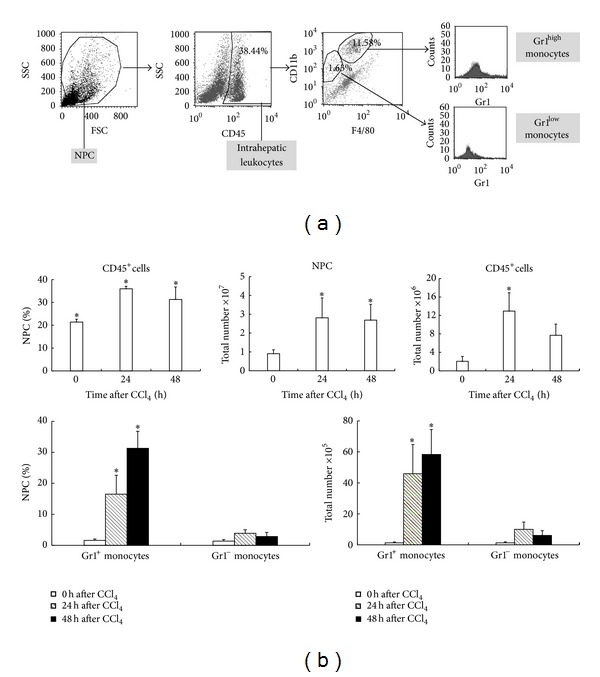
Gr1^hi^ but not Gr1^lo^ monocytes were massively recruited into CCl_4_-injured livers. (a) Flow cytometric analysis of intrahepatic monocytes. Non-parenchymal cells (NPCs) were isolated from CCl_4_-injured livers, and leukocytes were firstly gated on CD45^+^; then two monocyte subsets were distinguished based on CD11b and F4/80 expressions. The CD11b^+^F4/80^+^ subset (right upper gate) expresses high Gr1; the CD11b^+^F4/80^−^ population expresses low Gr1. The percentage of cells in selected gates is indicated. (b) NPCs were isolated from livers of different experimental groups at 24 h and 48 h after CCl_4_ injection. The total number of NPCs, the percentage and absolute number of CD45^+^ leukocytes in NPCs, and the percentage and absolute number of different monocyte subsets in NPCs were analyzed by flow cytometry using the strategy depicted in (a). Data are expressed as the mean ± (SD) (*n* = 8 mice/group). *P < 0.05 versus normal control (0 h).

To elucidate the effects of Mat on infiltration of different monocyte subsets following liver injury, we further isolated NPCs from livers of CCl_4_-challenged mice and characterized the different monocyte subset composition by flow cytometry analysis. As shown in Figure 2(a), the population of intrahepatic leukocytes was first defined as CD45^+^ cells. Using the myeloid marker CD11b and the macrophage marker F4/80 antigen, two distinct subsets of intrahepatic monocytes/macrophages could be identified: CD11b^+^F4/80^−^ and CD11b^+^F4/80^+^ cells. The CD11b^+^F4/80^+^ cells were found to express high level of Gr1, thereby resembling the phenotype of the peripheral Gr1^hi^ monocyte subpopulation. On the other hand, CD11b^+^F4/80^−^ cells expressed low levels of Gr1, corresponding to peripheral Gr1^lo^ monocytes (Figure 2(a)). This result is consistent with that in the previous report [18].

After isolation of NPCs from each liver, they were at first enumerated. The results in Figure 2(b) showed that CCl_4_-challenge led to significant increase in the total number of NPCs at 24 h and 48 h following CCl_4_ injection. Flow cytometry analysis revealed that both the percentage and the absolute number of CD45^+^ leukocytes were increased in the injured livers at 24 h and 48 h after CCl_4_ injection. Among the subsets of CD45^+^ leukocytes, the percentage of CD11b^+^F4/80^+^  Gr1^hi^ monocytes subset tremendously increased at 24 h and 48 h after CCl_4_ challenge, while CD11b^+^F4/80^−^ cells only mildly increased after CCl_4_ injection. According to the percentage of different subsets obtained in flow cytometry analysis and the total number of NPCs in each liver, the absolute numbers of different monocyte subsets were calculated. The result revealed that the absolute number of CD11b^+^F4/80^+^  Gr1^hi^ monocytes subset was dramatically elevated at 24 h and 48 h after CCl_4_ challenge, whereas that of CD11b^+^F4/80^−^  Gr1^lo^ subset only mildly increased (Figure 2(b)). This result demonstrated that the infiltration of the two monocyte subsets was differentially regulated following acute liver injury, and only CD11b^+^F4/80^+^  Gr1^hi^ subset massively increased after CCl_4_ challenge. 

**Figure 3:**
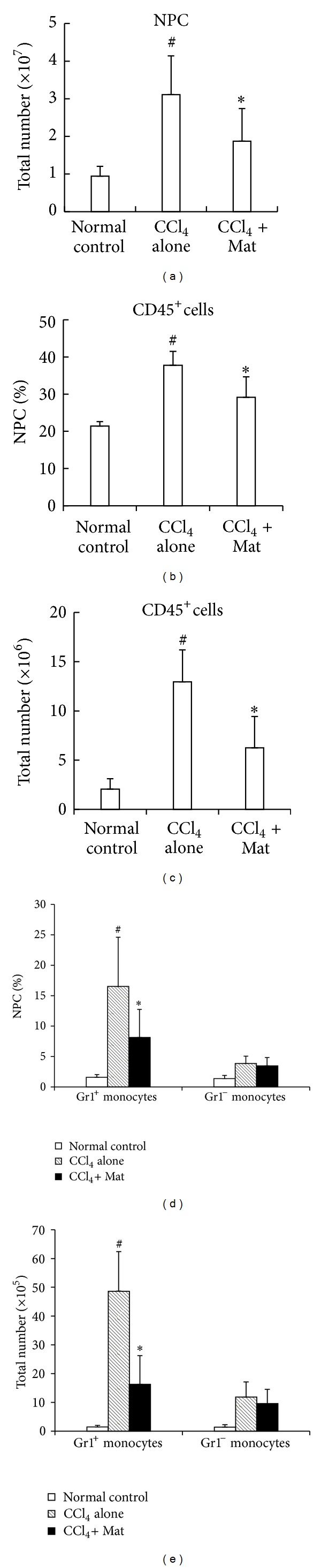
Mat inhibited the recruitment of Gr1^hi^ monocytes into acute liver injury. C57BL/6 mice were injected i.p. with CCl_4_ (0.6 mL/kg). Mat (30 mg/kg) was administrated orally 3 h before CCl_4_ injection. NPCs were isolated from livers of different experimental groups at 24 after CCl_4_ injection. (a) The total number of NPCs, (b) the percentage of CD45^+^ leukocytes in NPCs, (c) the absolute number of CD45^+^ leukocytes in NPCs, (d) the percentage of different monocyte subsets in NPCs, and (e) the absolute number of different monocyte subsets were analyzed by flow cytometry. Data are expressed as the mean ± (SD) (*n* = 8 mice/group). *P < 0.05 versus CCl_4_-only group; ^#^ *P* < 0.05 versus normal control group.

We next investigated the effects of Mat on the recruitment of Gr1^hi^ monocytes following acute liver injury. As shown in Figure 3, treatment with 30 mg/kg of Mat resulted in a significant decrease in the total number of NPCs (Figure 3(a)), as well as in the percentage and the absolute number of CD45^+^ leukocytes in NPCs, at 24 h after CCl_4_ challenge (Figures 3(b) and 3(c)). Furthermore, both the percentage and the absolute number of CD11b^+^F4/80^+^  Gr1^hi^ monocytes in injured livers were also significantly reduced by Mat treatment, while the infiltration of CD11b^+^F4/80^−^  Gr1^lo^ subset was almost not affected (Figures 3(d) and 3(e)).

### 3.3. Mat Reduces the Production of MCP-1

**Figure 4:**
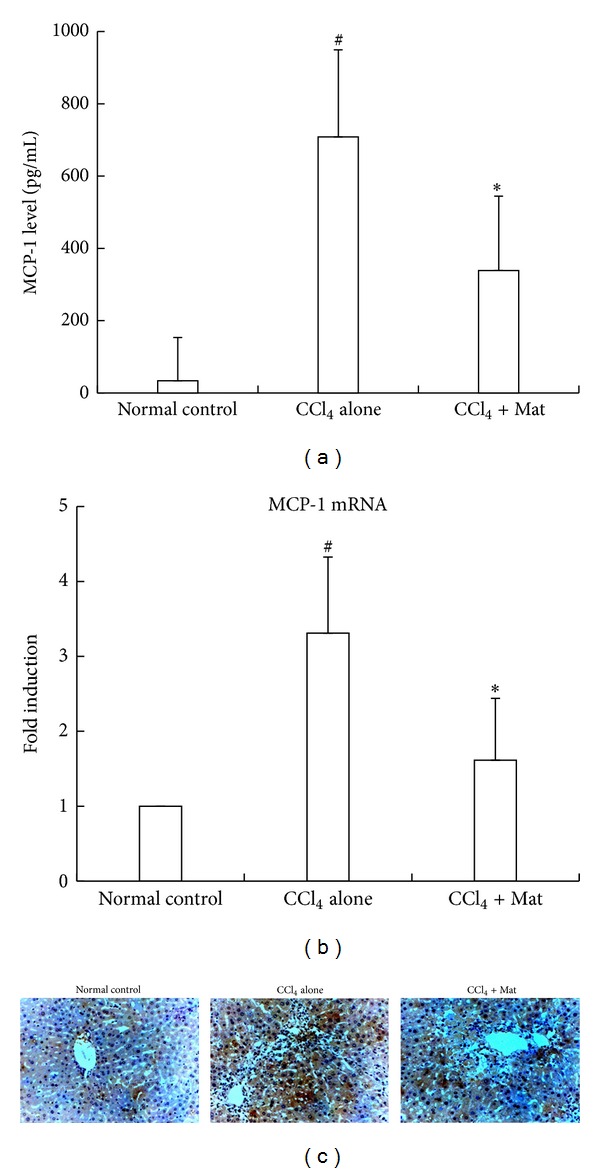
Administration of Mat reduced MCP-1 production in CCl_4_-challenge mice. C57BL/6 mice were injected i.p. with CCl_4_ (0.6 mL/kg). Mat (30 mg/kg) was administrated orally 3 h before CCl_4_ injection. (a) Sera were collected and analyzed for MCP-1 levels 24 h after CCl_4_ injection. (b) Liver tissues were collected 24 h after CCl_4_ injection and analyzed for MCP-1 mRNA levels with real-time PCR. Data in (a) and (b) are expressed as the mean ± (SD) (*n* = 8 mice/group). *P < 0.05 versus CCl_4_-only group; ^#^ *P* < 0.05 versus normal control group. (c) Liver tissues were collected 24 h after CCl_4_ injection; paraffin sections were made and analyzed for MCP-1 with immunohistochemistry (original magnification ×200).

The above finding that Mat selectively blocks the infiltration of Gr1^hi^ monocytes in CCl_4_-injured livers prompted us to investigate whether Mat could inhibit the production of MCP-1, a chemokine that crucially controls the recruitment of Gr1^hi^ monocytes. We found that serum MCP-1 was strongly upregulated following liver injury, and Mat treatment resulted in a significant decline of serum MCP-1 level in CCl_4_-challenged mice (Figure 4(a)). In line with the change in serum MCP-1 level, hepatic MCP-1 expression was strongly upregulated after damage as determined by real-time PCR and immunohistochemical analysis, which was also markedly decreased by treatment of Mat (Figures 4(b) and 4(c)).

**Figure 5:**
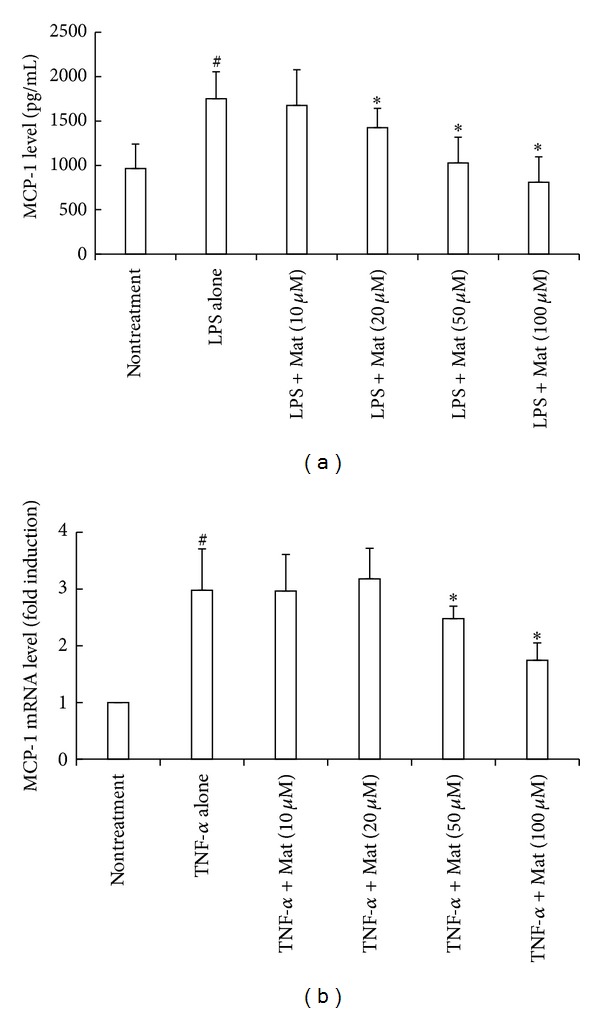
Mat inhibited MCP-1 production in NPCs and HSCs. (a) NPCs were isolated from CCl_4_-injured livers and stimulated with LPS (1 *μ*g/mL) for 24 h in the absence or presence of the indicated concentrations of Mat. MCP-1 levels in the supernatants were measured by ELISA. (b) HSCs were isolated from CCl_4_-injured livers and stimulated with TNF-*α* (30 ng/mL) for 24 h in the absence or presence of Mat. MCP-1 mRNA levels were measured by real-time PCR. Data were obtained from 3 independent experiments and presented as mean ± (SD). *P < 0.05 versus LPS or TNF-*α* alone;  ^#^ *P* < 0.05 versus nontreatment control group.

Since NPCs, especially activated HSCs, are major MCP-1-producing cells upon liver damage [21, 37–39], we further investigated whether Mat could directly inhibit MCP-1 production in these cells. The result showed that Mat (20–100 *μ*M) dose dependently inhibited MCP-1 secretion from LPS-stimulated NPCs isolated from CCl_4_-injured livers (Figure 5(a)). Mat also dose dependently suppressed the TNF-*α*-stimulated upregulation of MCP-1 mRNA in HSCs isolated from CCl_4_-injured mice in the range of 50–100 *μ*M (Figure 5(b)). 

### 3.4. Mat Inhibits the Chemotactic Activity of MCP-1

**Figure 6:**
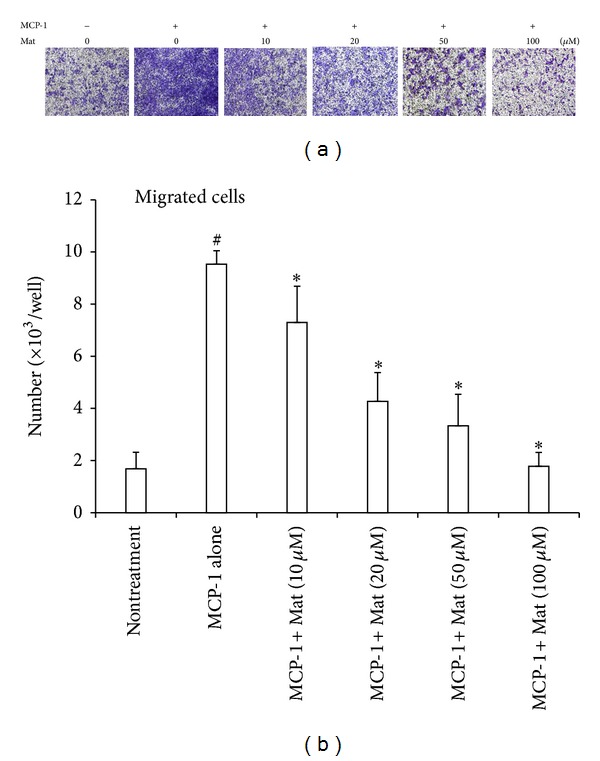
Mat inhibited the chemotactic activity of MCP-1. Chemotaxis assay was carried out with transwell culture chambers. Culture mediums containing 10 ng/mL MCP-1 were added to the lower wells of the chambers, and 1 × 10^5^ murine PBMCs were seeded into the upper wells in the absence or presence of the indicated concentrations of Mat. (a) After 2 h incubation, the cells migrating to the lower surface of the membrane were examined under microscope after being fixed with methanol and stained with crystal violet (original magnification ×100). (b) Four different areas of migrated cells were counted for each data point, and the number of migrated cells per well was calculated. Data were obtained from 3 independent experiments and presented as mean ± (SD).  *P < 0.05 versus MCP-1 alone;  ^#^ *P* < 0.05 versus nontreatment control group.

In addition to suppression of MCP-1 production, Mat may also act by inhibiting the chemotactic activity of MCP-1. So, we further determined this possible effect of Mat with transwell chemotaxis assays. As shown in Figure 6, MCP-1 apparently promoted the chemotactic migration of PBMCs, which can be inhibited by Mat in a dose-dependent manner in the range of 10–100 *μ*M (Figure 6).

### 3.5. Mat Exhibits Antifibrotic Effects and Inhibits Hepatic Infiltration of Gr1^hi^ Monocytes and Production of MCP-1 in Chronic CCl_4_-Challenged Mice

**Figure 7:**
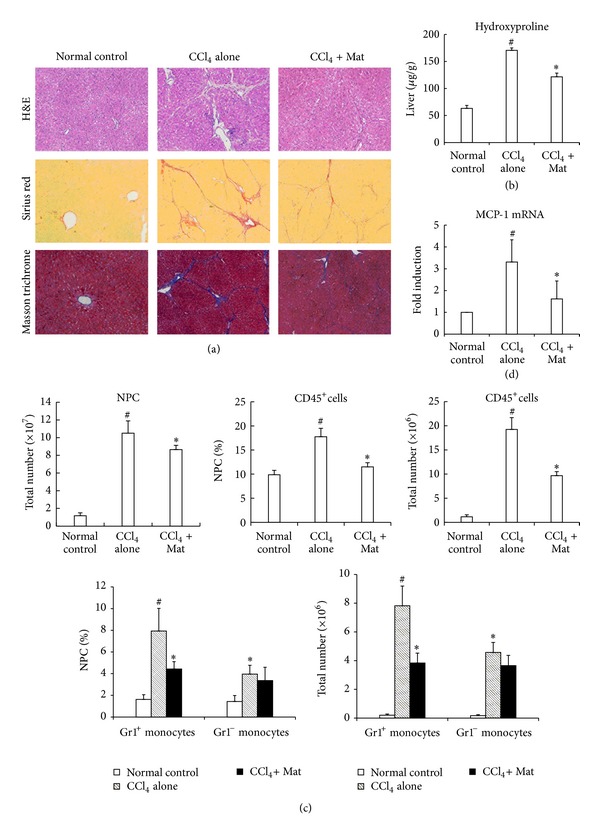
Mat exhibited antifibrotic effects and inhibited hepatic infiltration of Gr1^hi^ monocytes and production of MCP-1. C57BL/6 mice were injected i.p. with CCl_4_ (0.6 mL/kg, diluted in corn oil) or the same volume of corn oil twice weekly for 6 weeks. Mat (10 mg/kg) was orally administrated 5 times weekly for the last 3 weeks. Mice were sacrificed for analysis at 48 h after the last CCl_4_ injection. (a) Liver sections were stained with H&E, Sirius red, and Masson trichrome, respectively (original magnification ×100). (b) Liver hydroxyproline content was measured. (c) NPCs were isolated from livers, and the total number of NPCs, the percentage of CD45^+^ leukocytes in NPCs, the absolute number of CD45^+^ leukocytes, the percentage of different monocyte subsets in NPCs, and the absolute number of different monocyte subsets were analyzed with flow cytometry using the strategy as depicted in Figure 2. (d) Hepatic MCP-1 mRNA levels were analyzed with real-time PCR. Data in (b)–(d) are expressed as the mean ± (SD) (*n* = 8 mice/group).  *P < 0.05 versus CCl_4_-only group; ^#^ *P* < 0.05 versus normal control group.

The above experiments on acute liver injury model suggested that Mat could inhibit the development of liver fibrosis by blocking hepatic infiltration of Gr1^hi^ monocytes. We, therefore, examined its effects in a liver fibrosis model. Chronic administration of CCl_4_ twice weekly for 6 weeks resulted in significant collagen deposition and liver fibrosis in mice. Oral administration of Mat (10 mg/kg, 5 times weekly for the last 3 weeks) significantly reduced the accumulation of collagen and content of liver hydroxyproline in chronic CCl_4_-challenged mice (Figures 7(a) and 7(b)). Similar to the observations after acute injury, two subsets of monocyte-derived intrahepatic cells could be distinguished in the fibrotic livers. The CD11b^+^F4/80^+^  Gr1^hi^ subset was largely increased during fibrogenesis, while the CD11b^+^F4/80^−^  Gr1^lo^ subset only mildly increased. Administration of Mat significantly attenuated intrahepatic infiltration of Gr1^hi^ monocytes, but it did not influenced Gr1^lo^ subset (Figure 7(c)). Mat also inhibited intrahepatic expression of MCP-1 in chronic CCl_4_-challenged mice (Figure 7(d)). These results demonstrated that Mat prevented development of hepatic fibrosis and blocked recruitment of Gr1^hi^ monocytes into chronic injured livers through inhibiting MCP-1 production. 

## 4. Discussion

Mat has been demonstrated to be effective in suppressing inflammation in various inflammatory animal models [25, 28, 29, 31, 32, 40]. In particular, Mat has been shown to protect animals from acute liver injury induced by hepatotoxins and/or LPS [26, 41, 42]. Mat also exhibits anti-inflammatory and anti-fibrotic effects in CCl_4_-induced liver fibrotic models [30]. However, the underlying mechanisms still remain elusive. Since activation of KCs and HSCs has been well established as critical initial and relevant events in the development of liver fibrosis, we have previously tested the effects of Mat on these cells in an attempt to explore the mechanisms of Mat. Indeed, Mat has been found to inhibit TNF and IL-6 production from LPS-stimulated rat KCs [33] and to suppress serum- or PDGF-induced cell proliferation as well as serum- or TGF-*β*-induced collagen synthesis in rat HSC cell line HSC-T6 *in vitro* [30]. However, these pharmacologic activities are quite weak, as Mat only exerts significant inhibitory effects on the aforementioned cellular functions at concentrations higher than 250 *μ*M in those *in vitro* studies. In the present study, we try to identify other relevant cellular events that may be targeted by Mat to further elucidate the mechanisms underlying its anti-inflammatory and antifibrotic actions. 

It is well known that, during liver damage caused by different etiologies, activation of local cells is always associated with leukocyte infiltration from the bloodstream. In most cases, leukocyte infiltration results in damage amplification and generation of fibrogenic stimuli via secretion of soluble mediators and oxidative stress-related products [43]. Recently, infiltration of blood-derived macrophages has been suggested to be essential for liver fibrogenesis in addition to activation of liver-resident KCs [9, 44]. Furthermore, only the inflammatory Gr1^hi^ monocytes but not Gr1^lo^ monocytes are massively recruited into the injured livers following the acute and chronic challenge of CCl_4_, and they promote the progression of liver fibrosis, thus suggesting that Gr1^hi^ monocytes may represent an interesting target for anti-fibrotic strategies [18]. In the present study, we confirmed that liver injury, either acute or chronic, induced by single or repeated CCl_4_ injections, was associated with selective recruitment of Gr1^hi^ monocytes into livers. Furthermore, *in vivo *administration of Mat not only alleviated the acute liver injury induced by single CCl_4_ injection but also attenuated liver fibrosis in CCl_4_-induced chronic hepatic injury model. More importantly, Mat treatment significantly prevented the hepatic infiltration of the inflammatory Gr1^hi^ monocyte subset in livers in both acute and chronic liver injury models. Since intrahepatic Gr1^hi^ monocyte-derived cells have been previously demonstrated to differentiate preferentially into inducible nitric oxide synthase-producing macrophages during chronic liver damage and to exert proinflammatory and profibrogenic actions, such as promoting HSCs activation, TH1 differentiation and TGF-*β* release [18], our present finding suggests that inhibitory effect of Mat on the infiltration of Gr1^hi^ monocytes may contribute, at least in part, to its anti-inflammatory and antifibrotic effects.

Accumulating evidence indicates that recruitment of Gr1^hi^ monocytes into inflamed tissues is critically dependent on CCR2, a cognate receptor for C-C chemokine MCP-1 expressed mainly in monocytes [14–18, 45, 46]. A previous study using MCP-1 knockout mice has demonstrated that lack of MCP-1 affords protection from liver damage and development of oxidative stress in CCl_4_-induced acute liver injury model [47]. Another study using MCP-1-specific antisense phosphorothioate oligodeoxy nucleotides and specific CCR2 inhibitors has also shown that human peripheral CD14-positive monocytes contribute directly to organ fibrogenesis by an MCP-1/CCR2-dependent amplification loop [48]. A more recent study has further demonstrated that toxic liver damage results in a sequence of increased hepatic MCP-1 expression, elevated serum MCP-1, and peripheral blood monocytosis in wild-type mice. By contrast, CCR2-deficient mice lack peripheral blood monocytosis after injury and subsequently harbor significantly less Gr1^hi^ monocyte-derived intrahepatic macrophages [18]. In the present study, upregulation of intrahepatic MCP-1 expression following liver damage in both acute and chronic liver injury models was all significantly suppressed by *in vivo *administration of Mat, suggesting that Mat may inhibit MCP-1 production under inflammatory conditions. Cellular experiments using *in vitro *cultured NPCs and HSCs derived from CCl_4_-injured livers further demonstrated that Mat directly inhibited MCP-1 production in both LPS-stimulated NPCs and TNF-*α*-stimulated HSCs in a dose-dependent manner within the range of 20–100 *μ*M. These results corroborate the inhibitory effects of Mat on MCP-1 production induced by proinflammatory stimuli. In addition to the inhibition of MCP-1 production, Mat also exhibited the inhibitory effects on the chemotactic activity of MCP-1. In our transwell chemotaxis assays, Mat significantly inhibited MCP-1-mediated chemotactic migration of PBMCs dose dependently in the range of 10–100 *μ*M. Based on these data, we attribute the inhibition of Mat on hepatic recruitment of Gr1^hi^ monocytes to its inhibition of both MCP-1 production and its chemotactic activity. Yet, we cannot further explore the molecular mechanisms by which Mat exerts it inhibitory effects on MCP-1 production and function in the present study. Further study on the effects of Mat on the intracellular signaling involved in MCP-1 production and activity and identification of its molecular targets will help to fully elucidate the related mechanisms. In addition, even though it has been well documented that the recruitment of Gr1^hi^ monocytes into inflamed tissue is critically dependent on CCR2/MCP-1, other chemokines and their receptors are also involved in monocyte trafficking in some inflammatory settings, such as IL-8, CX3CL1, CCR5, and CX3CR1 [14, 20]. Therefore, the possible effects of Mat on production and function of these chemokines remain to be investigated to fully understand the mechanisms by which Mat inhibits the hepatic recruitment of Gr1^hi^ monocytes.

## 5. Conclusion

The present study demonstrates that *in vivo* administration of Mat affords protection from liver injury and development of liver fibrosis in CCl_4_-induced liver injury model, and such beneficial effects could be contributed, at least in part, to the prevention by Mat on the hepatic infiltration of the inflammatory Gr1^hi^ monocyte subset in injured livers, which is most possibly through its inhibition of both MCP-1 production and activity. These new findings extend our understanding on the mechanisms underlying its anti-inflammatory and antifibrotic effects.
